# Improved determination of plasmid copy number using quantitative real-time PCR for monitoring fermentation processes

**DOI:** 10.1186/1475-2859-7-6

**Published:** 2008-03-07

**Authors:** Mihaela Škulj, Veronika Okršlar, Špela Jalen, Simona Jevševar, Petra Slanc, Borut Štrukelj, Viktor Menart

**Affiliations:** 1 Lek Pharmaceuticals d.d., a Sandoz company, Verovškova 57, SI-1526 Ljubljana, Slovenia; 2 National Institute of Chemistry, Hajdrihova 19, SI-1000 Ljubljana, Slovenia; 3 Faculty of Pharmacy, Department of Pharmaceutical Biology, University of Ljubljana, Aškerèeva 7, SI-1000 Ljubljana, Slovenia

## Abstract

**Background:**

Recombinant protein production in *Escherichia coli *cells is a complex process, where among other parameters, plasmid copy number, structural and segregational stability of plasmid have an important impact on the success of productivity. It was recognised that a method for accurate and rapid quantification of plasmid copy number is necessary for optimization and better understanding of this process. Lately, qPCR is becoming the method of choice for this purpose. In the presented work, an improved qPCR method adopted for PCN determination in various fermentation processes was developed.

**Results:**

To avoid experimental errors arising from irreproducible DNA isolation, whole cells, treated by heating at 95°C for 10 minutes prior to storage at -20°C, were used as a template source. Relative quantification, taking into account different amplification efficiencies of amplicons for chromosome and plasmid, was used in the PCN calculation. The best reproducibility was achieved when the efficiency estimated for specific amplicon, obtained within one run, was averaged. It was demonstrated that the quantification range of 2 log units (100 to 10000 bacteria per well) enable quantification in each time point during fermentation. The method was applied to study PCN variation in fermentation at 25°C and the correlation between PCN and protein accumulation was established.

**Conclusion:**

Using whole cells as a template source and relative quantification considering different PCR amplification efficiencies are significant improvements of the qPCR method for PCN determination. Due to the approaches used, the method is suitable for PCN determination in fermentation processes using various media and conditions.

## Background

The production of recombinant proteins in *Escherichia coli *bacteria is affected by the number of plasmids, their structural and segregational stability, which have an essential impact on productivity. Maintenance of the segregational stability of plasmids is often a major problem during the fermentation process. The occurrence and enlargement of the population without plasmids due to low segregational stability usually leads to a significant loss of productivity. The high plasmid copy number is a prerequisite for balanced distribution of plasmids between daughter cells after cell division [1]. The addition of antibiotics into the growth medium is the simplest and most broadly used method to preserve a high number of plasmids in bacterial cells [2]. Although this is acceptable on laboratory scale, the broader use of antibiotics on an industrial scale is not desirable due to environmental pollution. Nevertheless, to ensure a high production of recombinant proteins, it is necessary to maintain an optimal plasmid copy number in bacterial cells. This level must be sufficient for the desired gene dosage effect, yet not so high that it induces a metabolic burden and loss of cell resources [3]. To better understand and to optimize the recombinant protein production process, the accurate and rapid quantification of plasmid copy numbers is essential.

Several different methods for plasmid copy number determination have been described in the literature. These can be divided into two major groups: The first group is based on an indirect plasmid copy number (PCN) determination, by measuring the activity of the reporter protein coded by the plasmid and normalized to the number of bacteria [4]. The other group of methods is based on direct quantification of plasmid and chromosomal DNA quantity followed by the calculation of the ratio between them. The latter include CsCl centrifugation [5], Southern blot hybridization [6], high performance liquid chromatography (HPLC) [7], capillary electrophoresis [8] and agarose gel electrophoresis [9]. DNA extraction is necessary for the majority of the methods and this can lead to inaccurate determination of PCN due to usually incomplete and irreproducible extraction and precipitation processes. Agarose gel electrophoresis is the most frequently used method to determine PCN. This technique allows whole cell lysate analysis without DNA extraction; however, it demands some extensive preparation. Analysing whole cell lysate on the gel can cause difficulties in results interpretation due to smears present on the gel. In native cells plasmids are present in multiple forms (supercoiled, open circle, linearized, monomers, dimers and multimers) and appearing of several bands on a gel can be a reason for problems in their accurate quantification. To overcome this problem, restriction endonucleases can be used to linearize all plasmids, however it is an extra step and additional possibility of entire error increase. In addition to staining the samples with harmful ethidium bromide, the gel only accommodates a small number of samples. Therefore treatment of larger number of samples obtained during the fermentation process is quite difficult, time consuming and labour intensive process. Furthermore, the method is of limited value for its high coefficient of variation, especially for low PCN values [10].

At the present time, the quantitative real-time PCR (qPCR) technique is widely used in many different fields requiring nucleic acid quantification. These include gene expression [11-13], pathogens loads [14-16] and traceability of genetically modified organisms in food and feed [17,18]. Methods have already been established for the determination of transgene or gene copy number integrated into the genomes of plants [19-21] and animals [22,23]. Recently, some authors [10,24-27] also described the application of qPCR for PCN determination in bacteria. The plasmids and chromosomes in bacteria are independent and self-replicating units in a cell. Crosa et al. [28] defined the PCN as the number of copies of plasmid present per chromosome in a bacterium. Rocha [29] reported that there could be up to six chromosomes in a bacterium during the intensive growth phase as a result of multiple openings of replication forks. Therefore, in the fermentation process, the determined PCN by qPCR is the ratio between numbers of amplicons lying on plasmids and chromosomes, in a moment of sampling. The common approach for qPCR analysis is the use of isolated and purified DNA, which causes great variability in PCN calculation arising from different efficiencies of plasmid and chromosomal extraction. This problem was highlighted by Providenti et al. [25] who tested the use of whole cells as a source of template, but obtained low amplification efficiency and underestimated the PCN and Carapuça et al. [27] who introduced whole cells lysate in quantification of PCN. In PCN determination, absolute quantification [10,24] and relative quantification [24-26] were used, but both had limitations. With the absolute method, differences in amplification efficiency between samples and standard curves were ignored. In the case of the relative quantification, the differences in amplification efficiencies between each amplicon used were not taken into consideration. Tao et al. [26] used the ΔΔCt method to determine fold changes of plasmid copy number, where the efficiency (E) is supposed to be 2 (100%) which is rarely true. Providenti et al. [25] applied the equation; PCN = E^-ΔCt ^where ΔCt is defined as the Ct value of the gene of interest subtracted from the Ct of a single copy gene (i.e., reference gene) and the actual efficiency (E) of amplification is determined by the dilution curve. The equation is adequate if the efficiency of amplification is the same for chromosome and plasmid. However, it has not yet been elucidated how small deviations in individual efficiency of amplifications can affect PCN. Carapuça et al. [27] used a different approach, combining relative and absolute quantification. In this case, reference material was prepared by spiking purified plasmid DNA with non-transformed cells and used as a standard curve. The difficulties of this approach are accurate preparation of reference material in the proper quantification range, especially for monitoring fermentation process on account of significant changes in bacterial density and PCN.

In the presented work, an improved qPCR method has been developed for PCN determination in the fermentation process. The aim has been to keep the quantity and quality of nucleic acids in cells unchanged until qPCR analysis and to assure accurate determination of PCN. In this respect, special attention has been paid to sample treatment immediately after sampling from the fermentor, omitting DNA extraction and applying minimal sample treatment as possible. We further focused on the development of calculation method and tested relative quantification, considering different amplification efficiencies for amplicons on plasmid and chromosome and evaluated reliability and reproducibility of results. Finally, any such quantification method has to be fast, easy to perform and suitable for a large number of samples assessed with minimal expense. It must enable handling of small volume of reactions as well as minimal labour and time should be required.

## Methods

### Bacterial strains, plasmid, cultivation, fermentation

#### Bacterial strain, plasmid

The optimized gene for hG-CSF (human granulocyte colony stimulating factor) was subcloned into pET 3a vector (Novagene), which contains an ampicillin resistance gene and is *Col*EI-like replicon. *E. coli *BL21(DE3) strain (Novagen) transformed with pET3a-hG-CSF plasmid was than used for the over expression of recombinant protein hG-CSF.

#### Media

LBP/amp100 medium: modified Luria-Bertani medium supplemented with 100 mg/L ampicillin (Sigma), where tryptone was replaced by phytone (Becton Dickinson), and LBPG/amp100 medium: modified Luria-Bertani medium supplemented with 100 mg/L ampicillin (Sigma) and 2.5 g/L glucose (Sigma) were used to prepare inocula and were also used to prepare the growth media of *E. coli*.

GYSP medium: 20 g/L phytone (Becton Dickinson), 5 g/L yeast extract (Becton Dickinson), 10 g/L NaCl (Sigma), 10 g/L glucose (Sigma) and trace elements (FeSO_4_·7H_2_O (40 mg/L), CaCl_2_·2H_2_O (40 mg/L), MnSO_4_·*n*H_2_O (10 mg/L), AlCl_3_·6H_2_O (10 mg/L), CoCl_2_·6H_2_O (4 mg/L), ZnSO_4_·7H_2_O (2 mg/L), NaMoO_4_·2H_2_O (2 mg/L), CuSO_4_·5H_2_O (1 mg/L), H_3_BO_3 _(0.5 mg/L)) and GYSP/amp100 medium: GYSP medium supplemented with 100 mg/L ampicillin (Sigma) were used for production of hG-CSF. 0.4 mmol/L IPTG (GoldBioTechnology) was used as an inducer.

#### Shake flask culture

0.4 mL of bacterial culture from a frozen working cell bank (stored at -70°C) was transferred aseptically to the 100 mL of LBPG/amp100 or LBP/amp100 medium. The inoculum was grown overnight at 25°C, 160 rpm in the incubator shaker to OD_600nm _3–5 and then transferred into a 500 mL Erlenmeyer flask containing 10 – 200 mL GYSP/amp100, LBPG/amp100 or LBP/amp100 medium at a ratio 1:20 and incubated for 18–24 hours at 25°C, 160 rpm in the linear incubator shaker. Induction was performed by addition of IPTG into the production media to a final concentration of 0.4 mmol/L.

#### Laboratory fermentation

Inoculum for laboratory fermentation was prepared in the same way as for the shake flask culture. Inoculum was transferred into the production medium GYSP or GYSP/amp100 supplemented with 0.4 mL of Antifoam 204 (Sigma) at a ratio 1:20. Fermentation was carried out in a 7 L Applikon fermentor at 25°C, 600 rpm and 0.5 vvm of air for 24–25 h. Induction was performed by addition of IPTG into the production media together with inoculum to a final concentration of 0.4 mmol/L.

### PCN determination by qPCR

#### Sample preparation for qPCR

Different treatments were tested for their ability to keep cells unchanged in terms of quantity and quality of nucleic acids until the qPCR analysis. Centrifugation of samples, heating samples at 95°C and 99°C for 10, 15, 20 min and freezing at -20°C were explored. The treatment which gained the lowest Ct (threshold cycle) values was chosen for further applications. The examination was performed twice, using cultures in two different media. The same amounts of cells were used in all treatments. We assumed that degradation of DNA in cell lysates and possible DNA loss using centrifugation would gain higher Ct values.

#### Design of primer sets for qPCR

The chromosome sequence of the *E. coli *strain K12 was obtained from the public database of the National Center for Biotechnology Information (NCBI). The plasmid sequence was obtained from the commercial provider of the expression plasmid (Novagen). It was assumed that the *E. coli *B strain, which was used in the present study, contained the same DNA sequence for the gene for DNA polymerase I as the *E. coli *K-12 strain. To check this presumption, we sequenced *E. coli *BL21(DE3) strain chromosome specific amplicon and proved 100% homology in nucleotide sequence between *E. coli *K-12 and B strain. PCR oligonucleotides specific for the plasmid and chromosome on non-coding regions were designed using the Primer Express 2.0. software (Applied Biosystems, Foster City, CA). The primers are specific for the amplification of a 94 bp long amplicon on the plasmid in the intergen region before the T7 promoter and an 87 bp long amplicon on the chromosome in the promoter region of the *E. coli *DNA polymerase I gene. Exact data of primers position on reported sequences are presented in Table 1. Primers were synthesized by Applied Biosystems.

**Table 1:** Primers for qPCR method for determination of PCN in bacterium *Escherichia coli *containing plasmid pET3a

Target	Primer sequence	Position and sequence accession number	Length	Tm
plasmid	5'-CGGTTGCTGGCGCCTAT-3'	703–721 pET 3a, Novagen	17 bp	83°C
	5'-ACCATACCCACGCCGAAA-3'	780–797 pET 3a, Novagen	18 bp	
chromosome	5'-GCGAGCGATCCAGAAGATCT-3'	4044875 – 4044894 *E. coli *K12, Accession number U00096	20 bp	75°C
	5'-GGGTAAAGGATGCCACAGACA-3'	4044941–4044961 *E. coli *K12, Accession number U00096	21 bp	

#### Sequencing of qPCR amplicon

The amplicon on the chromosome was amplified using TaqMan^® ^PCR Core Reagent Kit (Applied Biosystems). Amplicones were inserted to the pGEM plasmid vector using pGEM^®^-T Easy Vector System I (Promega). Plasmids were isolated using QIAprep Spin Miniprep Kit (Qiagen). Inserts were sequenced using pUC M13 forward 24 mer and reverse 22 mer sequencing primers (Promega) and BigDye^® ^Terminator v3.1 Cycle Sequencing Kit (Applied Biosystems). PCR products were purified using the DyeEx 2.0 Spin Kit (Qiagen). Hi-Di™ Formamide (Applied Biosystems) was added to dried samples. Sequence was determined using POP-6™ Polymer on ABI PRISM^® ^310 Genetic analyzer, (Applied Biosystems).

#### Real-time qPCR using SYBR Green dye

Real-Time PCR reactions were performed in 10 μL mixtures. The mixture for one reaction contained 1× SYBR Green^®^PCR Master Mix (Applied Biosystems) with ROX™ as a passive reference dye for real-time PCR and AmpErase uracil N-glycosylase to prevent carry-over contamination, 100 nmol/L of forward, 100 nmol/L of reverse primer, and 3 μL of sample (1 to 10^8 ^bacterial cells). Separate reactions were prepared for detection of chromosomal and plasmid specific amplicons, each in triplicate. In order to improve precision, volumes smaller than 9 μL were not pipetted. Therefore, the reaction mixtures for the triplicates including templates were premixed in 0.2 mL microtubes and then divided into three wells on an ABI Prism™ optical 384 well reaction plate. Finally, the plate was sealed with an adhesive cover (Applied Biosystems). Real-time reactions were run on an ABI Prism^®^7900 HT Sequence detection system (Applied Biosystems) using the following universal cycling conditions for all amplicons: 2 min at 50°C (UNG activation), 10 min at 95°C (AmpliTaq Gold DNA polymerase activation), followed by 45 cycles of 15 s at 95°C and 1 min at 60°C. At the end, a dissociation stage was added: 95°C 15 s, 60°C 15 s and 95°C 15 s. Cycle threshold (Ct) values were determined after automatic adjustment of the baseline and manual adjustment of the fluorescence threshold, using SDS 2.2 software (Applied Biosystems).

#### Plasmid copy number determination

After thawing, the samples (bacterial culture treated as described above), were serially diluted. Each time, three to five dilutions of sample were prepared within the dynamic range (100 to 100000 bacteria per well) of the assay. After the run completion background was automatically adjusted by SDS software and threshold was manually placed on the lower linear part of the amplification curves. Ct values were then automatically generated by software and exported to Excel for further analysis, since the SDS program does not enable relative quantification using different amplification efficiencies for different amplicones, what was the most appropriate in our case. In Excel average Ct and SD values of triplicates were calculated for each dilution. Dilutions where SD of Ct values was greater than 0.3 were not used for relative standard curve construction and PCN calculation. Relative standard curve was constructed placing the log value of the amount of bacteria (determined according to dilution) on the x axis and threshold cycles on the y axis. It was constructed for both chromosome and plasmid. To assure accuracy, the relative standard curve was determined from three to five dilutions and on condition that r^2 ^≥ 0.99. The slope of the relative standard curve was used for amplification efficiency (E) calculation following equation 1. If the E is 2 then the amount of PCR product exactly doubles with each cycle and efficiency expressed in percent is 100%.

$$\begin{array}{l} \text{E}=10^{\left(-1/\text{slope}\right)}\\ \text{E}(\%)=(10^{\left(-1/\text{slope}\right)}-1)\times 100 \end{array}$$

The plasmid copy number (PCN) was determined using equation 2, considering different amplification efficiencies (E) and Ct values for the two amplicons (chromosome-c and plasmid-p).

PCN = (Ec)^Ctc^/(Ep)^Ctp^

The PCN was calculated for all dilutions of each sample. These were then averaged and the SD was calculated.

#### Evaluation of the qPCR method

A melting curve analysis was performed and the Tm (melting temperature) specific for each amplicon was determined. The specificity of the primer sets was examined in each run for all reactions. Template-free control reactions (NTC) were used to check the possibility of primer dimer formation.

In order to determine the range of quantification, 10 fold serial dilutions were made with pure chromosomal and plasmid DNA, as well as bacterial culture in the stationary phase. Pure DNA ranging from 1 to 10^9 ^copies and bacteria ranging from 1 to 10^8 ^cells were put into the reaction. The internal positive control (IPC, Applied Biosystems) was used to exclude concentrations of samples causing inhibition. The least number of target molecules in the reaction which does not yet cause stochastic effects (denoted by an SD of Ct values between triplicates which does not exceed 0.3) was defined as the lower limit of quantification (LOQ). NTC reactions were run on each plate to assure reliable LOQ determination which needed to be at least five cycles above the Ct obtained for the NTC. In the determined quantification range, the amplification efficiency for the amplicons employed was calculated on the basis of linear regression curve and its slope using equation 1.

Mixtures of non-transformed cells and pure plasmid DNA were prepared at a certain ratio in order to confirm the suitability of the method for the determination of the PCN variation in fermentation. PCNs of up to 250 are expected in the fermentation process. The starting point, designated as 1:1 ratio, was set where the amplification curves overlap, presumed approximately the same number of plasmid copies and bacteria in reaction. Subsequent ratios were obtained by increasing the amount of plasmid DNA, and PCNs were calculated using the equation 3, including the amplification efficiency of plasmid (Ep) and Ct values (chromosome-c and plasmid-p).

PCN = Ep^(Ctc-Ctp)^

The following ratios were tested 1:10, 1:50, 1:75, 1:100, 1:125, 1:150, 1:200 and 1:250. Each ratio was prepared three times and the CV (%) was calculated using equation 4.

CV = average × 100/SD

In order to prove the reliability of the method, PCNs determined by qPCR, using five parallel shake flask cultures in stationary phase, were also verified by agarose gel electrophoresis performed as described by Projan et al. [9]. CV(%) was calculated using equation 4.

#### Application of the developed qPCR method for monitoring PCN in fermentation processes

PCN variations were examined in two fermentation processes at a growth temperature of 25°C, in the first, antibiotic was added to the medium, and in the second it was omitted. Samples were collected from the bioreactor in triplicate every hour for the first 4 hours and then every 2 hours for the next 20 hours (24 or 25 hours total).

The immediate sample treatment, the set up of the qPCR reactions and the PCN determination were all performed as described above. Additionally, the right range of sample dilutions had to be defined for each time point in the fermentation process to find Ct values in the quantification range suitable for the plasmid and chromosome. Cell growth and PCN increase during fermentation had to be considered. The PCN calculation using Ct values (chromosome-c and plasmid-p) and an average amplification efficiency of plasmid (Ep average) and an average amplification efficiency of chromosome (Ec average) for samples in one run was examined, using equation 5:

PCN = (Ec average)^Ctc^/(Ep average)^Ctp^

In cases where more than one plate was needed to assay all samples that belong to one fermentation, one sample was replicated on both plates and the PCN was calculated for both cases. The quantification of the PCN was repeated three times for each fermentation. Intra- and inter-assay real-time PCR variabilities were assessed based on CV (%) for Ct values as well as for determined PCNs using equation 4.

### Growth curve

Samples for growth curve construction were taken every hour for the first 4 hours and after that every 2 hours (24 or 25 hours total). Optical density was measured at 600 nm.

### SDS-PAGE

**Figure 1:**
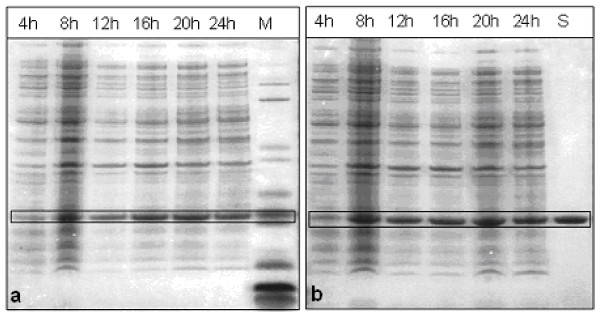
**Accumulation level of hG-CSF in fermentations at 25°C without ampicillin (a) and with ampicillin (b).** Escherichia coli BL21 (DE3) strain transformed with pET3a plasmid with gene for hG-CSF was used. Samples were taken every four hours, bands corresponding to hG-CSF are framed. M stands for Molecular weight standard Mark 12 with (Invitrogen) with purified hG-CSF; S stands for standard, purified hG-CSF.

Samples for SDS-PAGE analysis were taken every 2 hours. SDS-PAGE for protein detection was performed on 4–12% gradient gels using the NuPage Novex Bis-Tris SDS-PAGE (Invitrogen) system. SDS-PAGE gels were stained by SimplyBlue Safe Stain (Invitrogen) (Fig. 1). The accumulation level of hG-CSF in total cellular proteins was determined by densitometric analysis of SDS-PAGE gels stained by Colloidal Blue Stain Kit (Invitrogen) which enables quantification due to linear response of the proteins ranging from 0.1 – 2 μg. Densitometer model ProExpress Imaging System (Perkin Elmer) and TotalLab100 version 2006 software were used for the quantification of the accumulation level of hG-CSF.

## Results

### Sample preparation for qPCR

**Figure 2:**
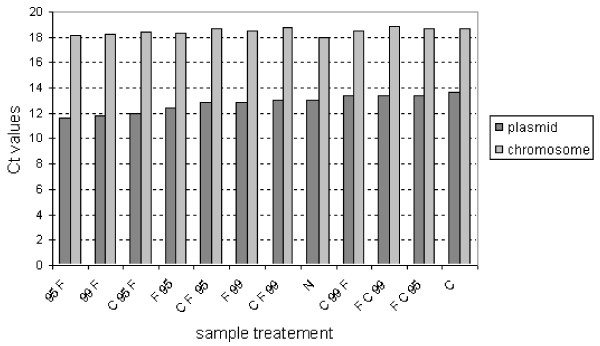
**Influence of different sample treatments on Ct values of plasmids and chromosomes in culture of *Escherichia coli *BL21 (DE3) containing pET3a plasmid with gene for hG-CSF from bioprocess in GYSP medium.** Sample treatments are marked as N nontreated, C centrifuged, F frozen, 95 and 99 temperature treatment in °C and are applied in subsequent steps as preformed. Duration of heating for 10 min, 15 min, 20 min at 95°C and 99°C are not presented separately.

As it is presented on Figure 2 the best treatment was the incubation of 1 mL of sample in a 1.5 mL microtube at 95°C for 10 minutes followed by immediate freezing at -20°C. This was proven in two separate trials. This treatment has to be carried out immediately after sampling from the bioprocess. It produced the lowest Ct values for plasmid and chromosome. Influence of different treatments on Ct value is greater for plasmid then for chromosome, what is reasonable because number of plasmids per cell is higher than number of chromosomes. Differences in Ct values for different treatments were in the range of 2.5 cycles for plasmid and 1 cycle for chromosome. Duration of heating for 10 min, 15 min and 20 min at 95°C and 99°C did not have influence on Ct values, therefore separate treatments are not presented on Figure 2.

The lowest Ct means that the highest copy number of targeted sequence is detected. Ct is dependent also on efficiency of amplification, better efficiency contributes to the lower Ct. Efficiency of amplification is high if DNA is pure and sample doesn't contain substances which inhibit PCR reaction. Different sample treatments have different impact on DNA. High temperature inactivates proteins and prevents degradation of DNA, weak centrifugation can lead to the cell loss when decanting supernatant, strong centrifugation to clotting the cells and therefore nonhomogenous samples. Low Ct value is measure that the maximal number of targeted sequence is detected and that sample does not contain inhibitors.

### Evaluation of the qPCR method

Sequencing of qPCR amplicons: Amplicons of *E. co*li strains K12 and BL21(DE3) shared a 100% nucleotide sequence identity.

Specificity of the qPCR assay: Data obtained by melting curve analysis of each reaction indicate that the amplification was specific. Each primer set gave a single sharp peak with a characteristic melting temperature of 83.7°C ± 0.5°C for the plasmid and 75.4°C ± 0.6°C for the chromosome. The mean value and the SD were calculated from fermentations samples data.

Range of quantification: The range of quantification for pure plasmid DNA was determined between 100 and 10^9 ^copies. For pure chromosomal DNA, this range was determined between 100 and 10^7 ^copies. A 3 log units quantification range was demonstrated for whole bacteria samples, to be between 100 and 100,000 bacteria. The method is valid for the PCN assessment of bacteria which have PCN up to 250. The amplification efficiency for whole bacteria samples in this range was around 1.81 (81%) for plasmid and around 1.95 (95%) for chromosomal DNA, calculated from fermentations samples data.

**Figure 3:**
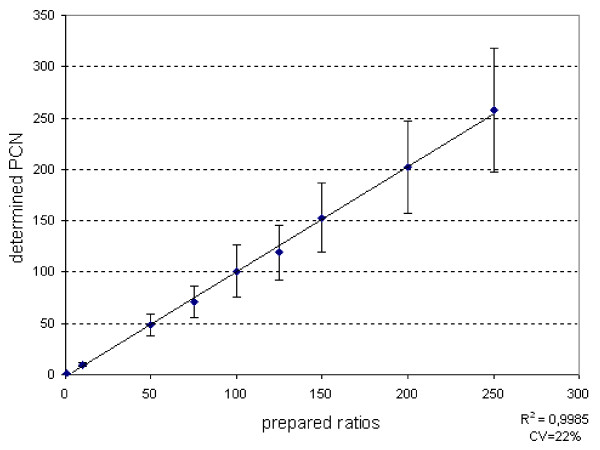
Determined PCNs for prepared ratios between non-transformed bacteria *Escherichia coli *strain BL21(DE3) and plasmids pET3a with hG-CSF.

Model systems: Determined PCNs of prepared mixtures between non-transformed bacteria and pure plasmid DNA in the ratios 1:1, 1:10, 1:50, 1:75, 1:100, 1:125, 1:150, 1:200, 1:250 are presented in Figure 3. The average CV for the determined PCN between three separately prepared samples of the same ratio was 23%.

Verification by agarose gel electrophoresis: Samples from five parallel shake flask cultures were used for development of the method. The average PCN value was 93 (intra-assay CV = 8%). Similar results were obtained with agarose gel electrophoresis, where the determined PCN was 104 (intra-assay CV = 12%).

### Application of qPCR method to fermentation

The development of this qPCR method revealed that calculating PCNs using a formula in which the amplification efficiencies for chromosomal and plasmid DNA are considered separately (equation 2) gave promising results. As shown in the next step, the variation in PCNs in a fermentation process can be better traced, with improved reproducibility of results, if the average amplification efficiency of all samples on one plate is used in the calculation (equation 5), rather than the specific amplification efficiency determined for each sample. Intra-assay (between dilutions of samples) and inter-assay variability for determined PCN were calculated for two fermentation processes at 25°C, with and without ampicillin in three time repetitions. In both processes, all the three repetitions had intra-assay variability in PCN from 7.39% to 9.97%. Inter-assay variability was 19.52% and 21.69%, which is in the same range as the variability obtained for separately prepared mixtures of different ratios of chromosomal and plasmid DNA. CV for Ct values of treated samples was under 0.8% for chromosome and under 0.5% for plasmid respectively.

### PCN variation in fermentation processes

**Figure 4:**
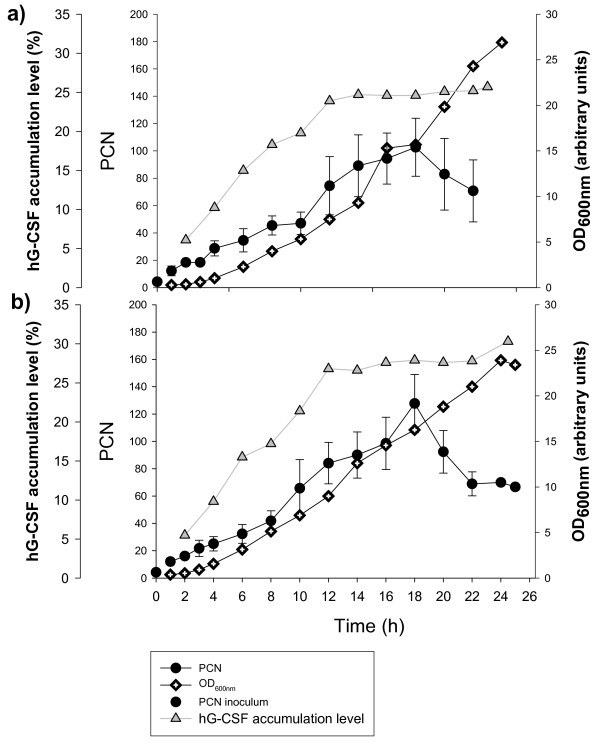
**Variation of PCN and hG-CSF accumulation level in fermentation. ***Escherichia coli *BL21 (DE3) strain transformed with pET3a plasmid with gene for hG-CSF was used. Fermentation was preformed in GYSP medium without ampicillin (a) and with ampicillin (b) at 25°C for 25 h.

PCN variations in fermentation processes were compared between a medium without ampicillin (Figure 4a) and another containing ampicillin (Figure 4b). In addition, the growth curve (OD) and hG-CSF accumulation level were presented. PCN increased significantly from the beginning of the process due to the addition of IPTG to the production medium, which causes metabolic stress [30]. The maximum PCN which was between 100 and 120 was achieved after 18 hours of cultivation, in the late exponential growth phase and is close to those expected (between 100 and 200). However a low copy number, less than 10, at the beginning of the process was not expected, since it is known, that copy numbers of *Col*EI-like replicons (which pET3a vector is) vary from 40–250 [31]. In the last 4 hours of cultivation, PCN decreased from the maximum to a value of 70. No significant difference was found between the two processes, ampicillin vs no ampicillin, for the calculated PCNs. As shown in Figure 4, the addition of antibiotic into the production medium also had no influence on the specific growth rate.

### Protein production

The recombinant human granulocyte colony-stimulating factor (hG-CSF) accumulation was monitored in the fermentation processes at 25°C in a medium with and without ampicillin (Figure 1). In the absence of antibiotic the maximum accumulation level was achieved after 12 hours of cultivation and it represented 25% of the total cellular proteins. In the presence of antibiotic, the maximum protein level was slightly higher and was around 28%. The accumulation levels did not decline afterwards. Figure 4 shows that PCNs increased from an initial value of 10 to a value of 80 after 12 hours of cultivation. Note that this peak in protein production does not coincide with the maximum PCN in the process. Unexpectedly, no significant difference was observed between the two experimental sets, which indicates the stability of the expression system. Since PCNs for processes with and without ampicillin are almost the same, it was expected that the addition of an antibiotic would not influence the protein accumulation level significantly.

## Discussion

Applicability of the qPCR method to PCN determination has recently been shown [10,24,25,25,27]. We tried to improve the qPCR method for an accurate PCN determination in the fermentation process. In the early stages of development, our method based on DNA isolation and absolute quantification, but this yielded very low reproducibility (data not shown), therefore another approach was chosen – using whole cells as a template source and consecutively relative quantification. Testing variants of this method showed that the most critical steps for reliable results were sample preparation and correct efficiency determination.

According to our experience and also based on the report of Friehs [1] and Carapuça et al. [27], the recovery of plasmid or total DNA when extracted or precipitated, is never 100%, regardless of the method used. Therefore, the determined PCN is always underestimated. For that reason we tried to avoid DNA isolation. However, to preserve the actual state of nucleic acids, we observed that the crucial step was the treatment of samples immediately after sampling from the fermentor. In this case, changes that can occur during storage, sample dilutions, reactions set up and analysis are avoided. It is desirable to employ as few steps as possible, because additional steps in the sample preparation process increase the variability of final determination. We proved that whole cell samples, underwent sufficient treatment, can meet the above listed requirements. For qualitative PCR analysis, the most frequently used treatment is lysis of bacteria by heating at 95°C for 10 or 15 min [32-34]. After heating, samples are usually further handled. In our case, additional steps such as centrifugation or/and freezing before or after heating failed. In contrast to Carapuça et al. [27] in our case immediate freezing of samples and lysis in the initial PCR denaturation step did not produce reproducible Ct values. Abolmaaty et al. [35] studied the effect of different lysis methods on the yield of *E. coli *DNA and its PCR amplifiability. The maximum yield of DNA was obtained after a combination of treatments with lysozyme, proteinase K and heating. However, the presence of either lysozyme or proteinase K in PCR reactions, even after their denaturation, partially inhibits the PCR reaction. Maximum amplification can be achieved only after complete lysis of bacteria and subsequent DNA purification. Heating of whole cell samples at 95°C for 10 minutes was proven to be an appropriate treatment in our case. However, it was understood that amplification was not perfect, which was considered in PCN calculation.

To reduce experimental error due to pipetting of whole cell samples, volumes smaller than 9 μL were not used. CV in Ct values was below 1% which was in the same range as was obtained for pure DNA.

When whole cells are used as template source, another issue associated with PCN quantification in fermentation processes, is the proper determination of the quantification range in order to avoid inhibition and stochastic variation. Clearly, these two effects are more pronounced when whole cells are used instead of pure DNA, as a result of the complexity of the samples and the size of bacteria. We found that at least 100-fold dilution of samples was enough to prevent inhibition. The possibility of inhibition should be checked in all experiments which employ different media. The stochastic effect does not appear if more than 100 cells are used per reaction. Furthermore, the method has to be adapted to a broad range of optical density of bacterial culture and broad range of PCN in order to be applied for monitoring a real fermentation process. The fermentation process starts at a low PCN (e.g., around 5–10 in the inoculum) and low OD in the early lag phase and ends up with a high PCN (e.g., 50–150 in the log phase) and high OD. This actually means that different sample dilutions have to be used for various parts of the bacterial growth curve, i.e. for low PCN samples one set of dilutions can be used and for high PCN samples another level of dilution has to be employed.

The calculation used in the presented study is only possible using SYBR Green detection chemistry since fluorescence generated by amplicons of about the same size is equal. There are some advantages in using SYBR Green detection chemistry as it can reveal the presence of primer dimers and non-specific products. Furthermore, SYBR Green is less expensive than alternatives and the costs are further lowered by adapting the assay to a 10 μL reaction.

In contrast to relative quantification for mRNA expression level determination where fold differences are important, relative quantification for copy number determination that enables detection of small differences in copy number poses a particular challenge. We affirmed the correct determination of amplification efficiency used for the PCN calculation is essential for an accurate PCN determination. The majority of currently applied qPCR methods assume that the target and reference gene amplify with similar efficiency in the same sample and also that differences between diverse samples are not significant. However, we employed a model where separate amplification efficiencies for reference and target gene were taken into account. This model was introduced for the determination of mRNA expression levels by Pfaffl [36]. Further, the model can be used for samples of bacterial culture from media consisting of different components. In our case, small differences in efficiency for target and/or reference gene generated substantial changes in PCNs and are intensified for higher PCN values (over 100). We found that an inaccurately determined PCR amplification efficiency of 5% for chromosome or plasmid misestimates PCN of 40 to 50%. If PCR amplification efficiency for both amplicons is changed equally in the same direction, no change occurs in the PCN value. An average amplification efficiency of more samples results in better estimation of real amplification efficiency, which significantly improves reproducibility of PCN determination.

The time-course of PCN values for our fermentation processes was similar to results that Carapuça et al. [27] observed for shake flask cultures. The maximum PCN was achieved in the mid to late exponential growth phase, which was also observed by Lee C.L. et al. [10] in their fermentation processes. A connection between PCN values and protein accumulation level has not been shown before. It was demonstrated that for maximum protein accumulation level, a maximum PCN value is not required, what is not unexpected result for pET expression plasmids.

## Conclusion

In this study, we have shown that the developed qPCR method can be a powerful tool for determination of PCN variation in fermentation processes under different cultivation conditions. Furthermore, we have shown the connection between PCN values and protein production. The use of whole cells as template source is big experimental advantage, since DNA isolation is avoided and a more realistic estimation of PCN is obtained.

Nevertheless, quantification should be done with caution and different amplification efficiencies for specific amplicons should be taken into account in order to achieve an accurate PCN quantification.

## Abbreviations

PCN plasmids copy number, qPCR quantitative real-time PCR, Ct threshold cycle, SD standard deviation, CV coefficient of variation, NTC no template control, E amplification efficiency, hG-CSF human granulocyte colony-stimulating factor, IPTG isopropyl β-D-1-thiogalactopyranoside, IPC internal positive control, LOQ limit of quantification.

## Authors' contributions

MS performed sample treatment, gel electrophoresis, qPCR and sequence analysis. VO participated in the design of the molecular genetics study and drafted the manuscript. ŠJa performed fermentations and SDS page. PS participated in the primer's design and performed some initial experimental work. SJe participated in the design of whole study (fermentation process, contributed the idea of using whole cells for PCN determination and was involved in selection of amplicon position) as well as in the drafting of the manuscript. BŠ and VM conceived the study, and participated in its design and coordination. All authors read and approved the final manuscript.
